# Measuring interprofessional competencies and attitudes among health professional students creating family planning virtual patient cases

**DOI:** 10.1186/s12909-016-0797-8

**Published:** 2016-10-19

**Authors:** Eric Wong, Jasmine J. Leslie, Judith A. Soon, Wendy V. Norman

**Affiliations:** 1 Faculty of Pharmaceutical Sciences, University of British Columbia, Vancouver, Canada; 2 Contraception Access Research Team- Groupe de recherche sur l’accessibilité à la contraception (CART/GRAC), Women’s Health Research Institute, British Columbia Women’s Hospital and Health Centre, Provincial Health Services Authority, Vancouver, BC Canada; 3 Department of Family Practice, Faculty of Medicine, University of British Columbia, 3rd Floor, David Strangway Building, 5950 University Boulevard, Vancouver, BC V6T 1Z3 Canada

**Keywords:** Sexual health education, Interprofessional, Health professional education, Medical education, Virtual patients, Medicine, Nursing, Midwifery, Pharmacy, Canada, Mixed methods, Survey, Qualitative

## Abstract

**Background:**

The Virtual Interprofessional Patients-Computer-Assisted Reproductive Health Education for Students (VIP-CARES) Project took place during the summers of 2010–2012 for eight weeks each year at the University of British Columbia (UBC). Undergraduate health care students worked collaboratively to develop virtual patient case-based learning modules on the topic of family planning. The purpose of this study was to evaluate the changes in perception towards interprofessional collaboration (IPC) among the participants, before and after the project.

**Methods:**

This study utilized a mixed methods evaluation using self-assessment survey instruments, semi-structured interviews, and reflective essays. Pre- and post- project surveys were adapted from the Canadian Medical Education Determinants (CanMEDS) and Canadian Interprofessional Health Collaborative (CIHC) frameworks, as well as the Memorial University Interprofessional Attitudes (IPA) questionnaire. The survey results were analyzed as mean (*M*) and standard deviation (*SD*) on Likert scales. The non-parametric Wilcoxon signed-rank test was used to determine if any significant changes were measured between each participant’s differences in score (*p* ≤ 0.05). Post-project interview transcripts and essays were analyzed using recursive abstraction to elicit any themes.

**Results:**

Altogether, 26 students in medicine, pharmacy, nursing, midwifery, dentistry, counselling psychology, and computer science participated in *VIP-CARES*, during the three years. Student attitudes toward IPC were positive before and after the project. At the project’s conclusion, there was a statistically significant increase in the participants’ self-assessment competency scores in the CanMEDS roles of *health advocate (p = 0.05)*, *manager (p = 0.02)*, and *medical expert (p = 0.03)*, as well as the CIHC domains of *interprofessional communication (p = 0.04)*, *role clarification (p = 0.01)*, *team functioning (p = 0.05)*, and *collaborative leadership (p = 0.01)*. Qualitative evaluations yielded three major themes: communication and respect as key to team functioning, importance of role clarification within the team, and existence of inherent challenges to IPC. From the reflections, students generally felt more comfortable with their improvements in the CIHC domains of *interprofessional communication*, *team functioning*, and *role clarification*.

**Conclusion:**

After working within an interdisciplinary team developing virtual patient learning modules on family planning, the student participants of the *VIP-CARES Project* indicated general improvement in the skills necessary for effective interprofessional collaboration. Triangulation of the overall data suggests this was especially observed within the areas of interprofessional communication, team functioning, and role clarification.

**Electronic supplementary material:**

The online version of this article (doi:10.1186/s12909-016-0797-8) contains supplementary material, which is available to authorized users.

## Background

In today’s medical system, delivering high-calibre health care often requires that professionals from different fields collaborate effectively [1]. Interprofessional collaboration (IPC) is defined as the process of developing and maintaining effective interprofessional working relationships with learners, practitioners, families, ﻿patients﻿, and communities to enable optimal health outcomes [2]. The World Health Organization has stated that IPC is essential in order to provide care for the people who are underserved by the medical system [3]. Furthermore, it has been recognized that this type of collaboration must begin early in the training of health care students to develop the necessary skills in this area [4]. For example, providers who infrequently interact with other disciplines during their education and training may encounter potential challenges in future collaborative work [5]. Recent literature suggests that there is a general positive attitude trend among health care students towards an acceptance of the principles of interprofessional education (IPE) [6, 7]. Therefore, it is beneficial to foster this type of collaboration among health care undergraduates.

The Virtual Interprofessional Patients-Computer-Assisted Reproductive Health Education for Students (VIP-CARES) Project was a student-led initiative, which took place during the summers of 2010–2012 for eight consecutive weeks each year. The project was developed by a group of medical, nursing, pharmacy, and midwifery students at the University of British Columbia (UBC) to address a curricular need after noting a lack of formal teaching in the topic of family planning. The aim of the *VIP-CARES Project* was to recruit an interdisciplinary team of health care students to work collaboratively in person on developing interactive virtual patient case-based learning modules in this area, for use in pre-licensure health professional training. Completed modules were integrated into their respective programs to fill gaps in curricula.

Currently, there is a lack of unity regarding the best methods to assess the professional outcomes of interdisciplinary learning projects [8, 9]. Despite the number of measurement instruments in IPC and IPE that have been published, only a few apply directly to health care teams [10, 11]. However, there are well-established IPC-related frameworks within and across health care disciplines that are often assessed. For example, the Royal College of Physicians and Surgeons of Canada developed a framework of professional competencies deemed essential for every practicing physician: the CanMEDS [12]. The competencies are comprised of seven roles: *communicator*, *collaborator*, *health advocate*, *manager*, *medical expert*, *professional*, and *scholar*. Not only has this tool been used to evaluate Canadian physician specialists in training [13], it is being used as a standard assessment tool to review competency in current physicians [14] in rural training sites [15], internationally [16, 17], and increasingly to assess a wide array of allied health care professionals [9].

The Canadian Interprofessional Health Collaborative (CIHC) also developed and published a framework that is specific for measuring interprofessional health care competencies [2]. The CIHC has outlined six major domains: *interprofessional communication*, *patient-centered care*, *role clarification*, *team functioning*, *collaborative leadership*, and *interprofessional conflict resolution*. There is much overlap between these domains and the CanMEDS roles. However, the CIHC framework specifically defines the skills and qualities necessary for effective IPC, whereas the CanMEDS more broadly defines the competencies required for overall patient care, a part of which includes IPC.

In addition to personal competencies, personal attitudes towards working in an interprofessional group can substantially impact the overall successful functioning of interprofessional teams. To assess these qualities, researchers from Memorial University developed the Interprofessional Attitudes (IPA) questionnaire [18]. This IPA questionnaire was developed by adapting three individual surveys related to attitudes towards IPE, each of which had previously been validated in their respective studies [19–21].

At the time of our study, the CanMEDS and CIHC frameworks, along with the IPA questionnaire, were the only established Canadian instruments relevant to evaluation of interprofessional attitudes and competencies. We utilized a combination of these three instruments for learner self-assessment, aiming to measure the development of attitudes and competencies for working in interprofessional teams. This study presents results from this survey among students participating in an interprofessional collaborative summer project, along with rich qualitative data providing depth, clarification, and substantiation of the quantitative results through findings from interviews and self-reflective essays. The purpose of this study is to evaluate the changes in perception towards interprofessional collaboration among pre-licensure health professional students, before and after working within an interdisciplinary team developing virtual patient case-based learning modules.

## Methods

This mixed methods study utilized a combination of self-assessment survey instruments to measure changes in interprofessional perceptions, including attitudes and competencies, before and after working on a collaborative, eight-week interprofessional project. Post-project interviews and reflective essays were completed by all participants. Ethics approval was obtained from the University of British Columbia (UBC), Children’s and Women’s Hospital Research Ethics Review Board (H10-00797) prior to the study.

Each participant completed surveys combining the CanMEDS and CIHC frameworks and the Memorial University IPA questionnaire (Additional file 1), to measure the self-assessed changes in interprofessional attitudes and competencies of the pre-licensure health professional students. The CanMEDS framework consists of seven roles, while the CIHC framework consists of six domains. The original IPA questionnaire from Memorial University is comprised of 42 questions across three components [18]. For our study, the fifteen relevant questions were selected within the components, *‘Attitudes towards health care teams’* and *‘Attitudes towards interprofessional education’*. Each question in the final evaluation tool asked participants to rank their expectations (pre-project) or experiences (post-project) on a 10-point Likert scale of *expectations met*, or on a 5-point scale of *level of agreement* to a statement. In addition, demographic information was collected regarding age, gender, discipline, year of study, and prior interprofessional experience(s). Prior interprofessional experience was defined as any team collaboration involving more than one health discipline, which took place before the start of the *VIP-CARES Project*.

During 2010–2012, three different cohorts (one each summer) of interdisciplinary health professional students from UBC worked together for eight consecutive weeks on the *VIP-CARES Project*. The purpose of the *VIP-CARES Project* was for the students to work collaboratively and in person on an interdisciplinary team to develop virtual patient case-based learning modules on family planning topics for implementation into their respective program curricula. Project activities included conducting team interviews with community family planning health service professionals from all project disciplines to learn about practice relevant perspectives and cases; collaborating to collect, evaluate, and integrate appropriate case and resource material; and utilizing the information to develop the virtual cases. Altogether, there were nine different virtual patient cases developed by the students (approximately three cases from each cohort).

The project was offered to students enrolled in any year of a health professional pre-licensure training program at UBC (including up to one year after graduation), as a paid summer work experience. Positions were also offered to computer science students for their expertise in human-computer interaction and the interfaces necessary for effective online self-learning modules. There were many more students who applied each year than there were positions available; each final cohort of students was selected through a panel of student interviewers who were rigorously involved in the early project development. Altogether, there were three different cohorts of students, each cohort combined students from the programs of medicine, pharmacy, nursing, midwifery, dentistry, counselling psychology, and computer science. The project was approved and funded by the UBC Teaching and Learning Enhancement Fund for each of the three years.

### Data collection

*VIP-CARES Project* students received a letter explaining the purpose and methods of this study, and a consent form two weeks before the project began.

Surveys were completed on the first and final days of each project. Following completion of the final survey, participants wrote a short, reflective essay of up to 500 words, and completed a semi-structured interview with a trained research assistant, in which they were asked to describe how their perceptions of IPC changed over the course of the *VIP-CARES Project*. The interviews were audio-recorded, professionally transcribed, and thematically analyzed as detailed below. The essays and interviews expanded upon and explored in more depth the concepts and attitudes introduced in the quantitative measures for interprofessional competencies. Results from the surveys, interviews, and reflective essays were linked to each participant, such that triangulation of themes and ideas could be evaluated.

### Statistical analysis

The results from the surveys were analyzed as mean (*M*) and standard deviation (*SD*) scored on a Likert scale from 1 to 5 (IPA), or from 1 to 10 (CanMEDS and CIHC). Both the mean and the standard deviation of the differences in score (*M*_*d*_, *SD*_*d*_) between each participant’s pre- and post- responses were also calculated. These differences were evaluated using the non-parametric Wilcoxon signed-rank test to determine if any significant changes were measured between each participant’s differences in score (significance level *p* ≤ 0.05). Interview transcripts and essays were analyzed using recursive abstraction to elicit themes and changes in attitudes or competencies over the course of the project. Themes from the qualitative data were elicited by two research assistants, then the categories were reviewed and any discrepancies resolved by two faculty investigators. Triangulation of self-assessment surveys, interviews, and essays within and between individual participants was performed to enhance the depth of understanding related to feedback from each of the student experiences and from the participants overall.

## Results

### Demographics

Table 1 describes the study participants. There were a total of 26 participants among 28 project students throughout the three-year project. The two students were excluded from the study because they were post-graduate medical residents who had completed their undergraduate programs beyond one year of their project enrolment. The average age of participants was 26.0 (±2.8) years old, and the female- to- male ratio was 23- to- 3 (88 % female). About half (54 %) had ‘prior interprofessional experience’.

**Table 1:** Demographic information of participants across the 3 years (2010–2012) who completed all components of the mixed methods assessment for the interprofessional *VIP-CARES Project*

Total number of students	26						
Mean Age	26.0 (±2.8)						
Gender	Female: 23 (88 %)						
Discipline	Medicine	Pharmacy	Nursing	Midwifery	Dentistry	Counselling Psychology	Computer Science
	(*n* = 7)	(*n* = 6)	(*n* = 4)	(*n* = 4)	(*n* = 1)	(*n* = 1)	(*n* = 3)
• Year 1 (2010) (9 participants)	3	2	2	2	0	0	0
• Year 2 (2011) (9 participants)	2	2	2	0	1	0	2
• Year 3 (2012) (8 participants)	2	2	0	2	0	1	1
Proportion of program years completed per discipline (*M*, *SD*)	0.36 (0.20)	0.75 (0)	0.63 (0.25)	0.38 (0.14)	0.25 (0)	1.0 (0)	1.0 (0)
Students with prior inter-professional experience per discipline	4	3	2	3	0	0	2

### Surveys

Table 2 summarizes the analysis of the CanMEDS, CIHC, and IPA sections within the administered surveys.

**Table 2:** Pre- and post- mean scores and differences on survey scales for interprofessional collaboration and competencies among *VIP-CARES Project* students

Interprofessional (IP) Attitudes section adapted from Memorial University Questionnaire (Five point Likert Scale: 1 = Strongly Disagree, 5 = Strongly Agree)	Mean score of all participants before *M* (*SD*)	Mean score of all participants after *M* (*SD*)	Mean score differences for each participant *M*_*d*_(*SD*_*d*_)	*p*-value
Number of completed surveys	*n* = 26	*n* = 26		
IP learning will help students to understand their own professional limitations.	4.4 (0.6)	4.5 (0.6)	0.0 (0.7)	1.00
Developing an IP patient/client care plan is excessively time consuming.	2.6 (0.8)	2.3 (0.8)	−0.3 (0.9)	0.11
The IP approach makes the delivery of care more efficient.	4.2 (0.7)	4.2 (0.5)	0.0 (0.6)	0.74
Developing a care plan with other team members avoids errors in delivering care.	4.2 (0.7)	4.3 (0.7)	+0.2 (0.7)	0.20
Working in an IP manner unnecessarily complicates things most of the time.	1.8 (0.5)	1.8 (0.6)	+0.1 (0.8)	0.44
The IP approach improves the quality of care to patients/clients.	4.6 (0.5)	4.3 (0.7)	−0.3 (0.7)	0.02^a^
In most instances, the time required for IP consultations could be better spent in other ways.	2.0 (0.7)	2.0 (0.7)	−0.2 (0.9)	0.44
IP approach permits health professionals to meet the needs of family caregivers as well as patients.	4.0 (0.6)	4.0 (0.7)	0.0 (0.9)	0.83
Team meetings foster communication among team members from different disciplines.	4.1 (0.6)	4.4 (0.6)	+0.2 (0.9)	0.18
IP learning will help students think positively about other health care professionals.	4.3 (0.6)	4.3 (0.8)	0.0 (0.8)	0.81
Clinical info can only be learned effectively when taught within one’s own department.	1.9 (0.9)	1.8 (0.6)	−0.1 (0.7)	0.74
Students in my professional group would benefit from IP small group projects.	4.2 (0.8)	4.2 (0.7)	+0.1 (0.7)	0.37
It is not necessary for undergraduate health care students to learn together.	1.8 (0.7)	1.7 (0.7)	−0.1 (0.6)	0.48
IP work before qualification would improve working relationships after qualification.	4.2 (0.6)	4.2 (0.9)	0.0 (1.0)	0.78
IP work helps undergraduates to become more effective team members.	4.3 (0.6)	4.3 (0.9)	0.0 (1.1)	0.64
*Competencies from the CanMEDS section (Ten point Likert Scale: 1 = Below Expections, 10 = Exceptional)*				
Number of completed surveys	*n* = 26	*n* = 26		
Communicator	6.4 (0.6)	6.7 (1.2)	+0.3 (0.9)	0.12
Collaborator	6.7 (1.2)	6.9 (1.1)	+0.3 (1.0)	0.18
Health Advocate	6.2 (1.2)	6.8 (1.3)	+0.5 (1.1)	0.05^a^
Manager	5.6 (1.5)	6.3 (1.3)	+0.6 (1.1)	0.02^a^
Medical Expert	5.0 (1.1)	5.7 (1.4)	+0.6 (1.3)	0.03^a^
Professional	7.3 (1.4)	7.2 (1.1)	−0.1 (0.9)	0.64
Scholar	6.3 (1.3)	6.8 (1.5)	+0.4 (1.6)	0.19
*Competencies from the CIHC section (Ten point Likert Scale: 1 = Below Expections, 10 = Exceptional)*				
Number of completed surveys	*n* = 22	*n* = 26		
Interprofessional Communication	6.2 (1.2)	7.0 (1.2)	+0.7 (1.6)	0.04^a^
Patient Centered Care	6.5 (1.2)	6.9 (1.5)	+0.4 (1.3)	0.24
Role Clarification	6.0 (1.3)	7.0 (1.3)	+0.9 (1.3)	0.01^a^
Team Functioning	6.3 (1.3)	7.2 (1.5)	+0.8 (1.7)	0.05^a^
Collaborative Leadership	5.9 (1.1)	6.9 (1.4)	+0.9 (1.4)	0.01^a^
Interprofessional Conflict Resolution	5.7 (1.5)	6.2 (1.6)	+0.6 (1.5)	0.10

^a^Statistically significant result

Overall, the participants responded with a positive attitude towards IPC in the IPA section of the survey, both before and after the *VIP-CARES Project*. However, participants less strongly agreed with the statement: *“the interprofessional approach improves the quality of care to patients/clients.”*

There was a trend towards higher participant self-assessment scores for the CanMEDS competency roles of *communicator*, *collaborator*, *health advocate*, *manager*, *medical expert*, and *scholar* post-project, as compared to baseline. Statistical significance was observed for the roles of *health advocate (p = 0.05)*, *manager (p = 0.02)*, and *medical expert (p = 0.03)*.

There was a similar trend towards higher participant self-assessment scores for all six CIHC competency domains: *interprofessional communication*, *patient-centered care*, *role clarification*, *team functioning*, *collaborative leadership*, and *interprofessional conflict resolution* post-project, as compared to baseline. Statistical significance was observed for the domains in *interprofessional communication (p = 0.04)*, *role clarification (p = 0.01)*, *team functioning (p = 0.05)*, and *collaborative leadership (p = 0.01)*.

### Qualitative findings

The qualitative findings comprising of the semi-structured interviews and the reflective essays supported and provided further explanation for the results from the surveys in most roles and domains. Most comments reflected positively on IPC and the *VIP-CARES Project* overall. Several consistent themes emerged from the post-project qualitative findings:

#### Communication and respect as key to team functioning

The most significant theme which emerged is how the qualities of communication and respect are considered to be central to successful IPC. Several participants commented on the importance of a committee, rather than a hierarchal structure within the group to help facilitate these qualities. The participants agreed that this created open communication between team members, and allowed for the sharing of ideas in a comfortable setting that was conducive to collaboration. For most participants, respect encompassed being receptive towards ideas which emerged within the group, regardless of each other’s interdisciplinary backgrounds. For example, one student spoke on the need to *“respect each other’s opinions throughout the project…and wanting to learn about each other and not having that feel [sic] like your profession is better than theirs.”*

#### Importance of role clarification within the team

Another theme was the importance of role clarification in an interprofessional setting. Most participants commented that the interprofessional roles of themselves and team members were more defined following the project. One student mentioned that *“when [the roles are] laid out, it leads to the health care providers having more satisfaction about what they’re doing and I think it comes off a bit clearer for the client too.”* By having an understanding of each other’s roles, the team members felt they were better able to contribute their specific areas of expertise on a particular subject, which may also extend into future career-related IPC. For example, a medical student commented on what she gained from working with a midwifery student: *“if someone were to ask me before the program started what exactly does a midwife do, I could sort of guess but I had no idea of how broad it was… [it] helps you to provide in the future…better maternity care service for patients for letting them know what’s out there as well.”* Several participants commented on learning about expanding scope of practice for other disciplines. Where there were areas of overlap between disciplines, the participants perceived it as being beneficial, rather than detrimental to team collaboration, as long as they were aware of where this overlap existed.

#### Existence of inherent challenges to interprofessional collaboration

A final theme which emerged is recognizing the potential inherent challenges to IPC. One participant wrote in her reflective essay how the project *“strengthened and emphasized the importance of interprofessional work in patient management”*; however, this same individual reflectively commented on how different personalities and agendas of team members can hinder collaborative productivity, noting that *“interprofessionalism is not as easy to accomplish as it sounds.”* Similar sentiments were noted by other participants, many of whom felt that a difficult personality in a team setting could hinder the progress of collaboration.

It is of interest that there were several students who were still uncomfortable about their abilities to apply these roles in future settings. As one student mentioned, *“I’m not far along enough in my training…like, to be able to advocate for my field of health care…I just don’t know enough about my field yet to be able to do that.”* Other students also commented that they expect their health advocacy to improve once they are out in practice. Students who commented on their change in the role of medical expert believed they were able to develop a patient-centered approach to decision-making and learn the limits of their expertise. However, others believed that they are *“not familiar enough in [their own] profession[s] at this stage to be considered an expert.”*

## Discussion

The *VIP-CARES Project* was undertaken during the early development stage of interprofessional collaboration for pre-licensure health professional students in Canada. Each team of interdisciplinary students worked collaboratively to create virtual patient case-based learning modules over the course of eight weeks. These self-learning cases on family planning topics have since been adapted into the health professional training curricula at UBC. In 2011, the project was also recognized with the national Health Innovation Award by the Health Council of Canada for the university-based practice in health care education most deserving to be a model for the rest of Canada [22].

Following the three eight-week interprofessional *VIP-CARES Projects*, student participants reported a self-assessed increase in competencies as measured by the frameworks of CanMEDS and CIHC. Our results show some promising trends. The CIHC framework specifically defines the skills and qualities necessary for effective IPC. We found significantly higher post-project scores as compared to the baseline surveys in the CIHC competency domains in *interprofessional communication*, *team functioning*, and *role clarification*, and that improvements in these areas were supported by the themes from the qualitative findings. Expanding on the survey results, we observed among the qualitative responses that participants generally felt more comfortable with their improvements within CIHC interprofessional competencies, as compared to the CanMEDS individual professional competencies of *health advocate*, *manager*, and *medical expert*. Similarly, for each participant, the mean score differences in the CIHC domains were higher (close to one full point), as compared to the mean score differences in individual achievement measures of the CanMEDS roles (close to half a point). This finding may reflect the predominance of pre-licensure students early in their professional careers disinclined or less confident to comment on their growth in individual professional competency areas. Attitudes towards IPC were positive amongst students before and after participating in the *VIP-CARES Project*. The participants’ IPA scores for attitudes towards interprofessional health care teams and education were high at baseline. These responses did not change significantly at the project’s conclusion.

The participants identified in the post-project semi-structured interviews and reflective essays that communication and respect were two important attributes to successful interprofessional work. These findings are closely associated with the CIHC competencies of *interprofessional communication* and *team functioning*, both of which were scored significantly higher by participants, as compared to baseline. Several participants noted that “*communicating and asserting themselves”* with other health care students was a weakness prior to starting the *VIP-CARES Project*. These participants later commented on an increase in their level of confidence when communicating with other team members, while remaining respectful of group diversity. During the project, each group of students had the opportunity for extensive interaction on an interdisciplinary team for eight consecutive weeks, which likely helped to facilitate IPC, as they were working towards a shared goal. Interestingly, the results from the interviews and essays did not convey any major themes regarding the perceived dominance of a particular health care profession. This was surprising, given that there were a disproportionately higher number of students from certain programs (i.e. medicine and pharmacy), as compared to others (i.e. dentistry and counselling psychology) each year. A possible explanation for this is the fact that the participants responded with a positive attitude towards IPC in general, both before and after the *VIP-CARES Project*. Although some participants related that there are inherent challenges to achieving IPC, this was more in the context of individual personality dynamics. It should also be noted that the nature of the project required regular, daily collaborative interaction over an eight-week period, which is not necessarily indicative of all interprofessional settings.

The responses to the CIHC section of the survey, along with the themes elicited from the reflective essays, support a general improvement in *role clarification* among the students. Participants seemed to gain a better understanding of their own roles, as well as the roles of other disciplines within the team. They reflected that this knowledge can be utilized to enhance patient/client/family goals, which may be especially important in family planning, as it is increasingly delivered within interdisciplinary team settings [23]. As the *VIP-CARES Project* focused on developing educational tools for family planning, the role of each team member was well-defined, as required for the development of specificity and complexity within the various virtual cases. This potentially allowed for greater role clarity over the course of the project, as participants were able to closely observe team members modeling their respective roles.

The higher scores in the CanMEDS roles of *health advocate*, *manager*, and *medical expert* from the surveys were generally supported by the themes from the interviews, but this was not as closely observed with the CIHC competencies. The concept of the *VIP-CARES Project* created ample opportunities for students to make achievements in these CanMEDS roles. For example, several module cases were based on challenges in caring for patients within at-risk or marginalized populations. In designing these cases, participants were guided to consider the determinants of health, including the physical, cultural, and economic barriers faced by vulnerable individuals in these populations. These skills are encompassed in both the CanMEDS *health advocate* and *medical expert* roles. Using information technology, as well as setting priorities and practicing time management skills are associated with the CanMEDS *manager* role [12]. The majority of the students had most recently completed the first or second years of their respective programs, with the exception of all pharmacy students who had completed their third year. Thus, at the end of the project, students appeared to still retain a level of uncertainty about their professional expertise, which appeared consistent with the current progress in their respective programs.

With regards to the attitudes towards interprofessional health care teams and education, participants less strongly agreed with the statement: *“the interprofessional approach improves the quality of care to patients/clients,”* at the end of the project. The mean IPA score for all participants for this question at baseline was 4.6 ± 0.5, which was resoundingly the highest baseline score within this section of the survey. Considering that the attitudes were ranked on a 5-point Likert scale and the mean score difference within each participant for the question was −0.3 ± 0.7, this level of change, although statistically significant, is unlikely to represent a clinically significant attitude shift. It may, however, more likely reflect a loss of pre-project anticipatory enthusiasm. Notably, no negative opinions related to *“the interprofessional approach improves the quality of care to patients/clients”* were documented in the qualitative findings.

Overall, the results of this study have various implications. The current literature suggests that there is a discordance regarding the best methods for assessing the professional outcomes of interdisciplinary learning projects [8, 9]. This study is the first to combine the IPC-related frameworks from established Canadian instruments relevant to evaluating interprofessional attitudes and competencies: CanMEDS, CIHC, and the IPA questionnaire. By applying this tool in the evaluation of the interprofessional development of virtual patient case-based learning modules, we were able to observe some meaningful results, which were supported by participant interviews and self-reflective essays. Future studies to validate this combined survey instrument are recommended. Furthermore, the *VIP-CARES Project* helped to further our understanding of how pre-licensure health professional students work collaboratively on a project of this level of regular, daily interprofessional interaction. This study also fills a gap in literature regarding the lack of interprofessional student collaboration in antenatal and postnatal care [24]. At the time, it fulfilled a curricular need at UBC, in providing more formalized teaching in the topic of family planning. Educational technology, in the form of virtual patients, is becoming more commonly used in medical education for its role in promoting deep learning and enhancing clinical reasoning skills [25]. However, they are frequently developed by medical educators in their respective health disciplines. The *VIP-CARES Project*, to our knowledge, is one of the first to allow for interdisciplinary students to work collaboratively on developing virtual patient cases, for implementation across multiple health care curricula.

Our results have implicit limitations. First, this study had a small sample size of 26 participants, with no more than 7 participants in each professional group (i.e. medicine). The reason for this limited size is due to the funding allocated from the UBC Teaching and Learning Enhancement Fund for each of the three years. Each student also received a stipend over the summer for their participation. Furthermore, the survey instrument used self-assessment as a means of measurement, which is inherently subjective. We noted unexpectedly high ratings of positive interprofessional attitudes and competencies at baseline, which likely reflected a bias in our sample. Project participants were selected from among a large, competitive group of pre-licensure health professional student applicants. In general, the participants were individuals who had sought out prior interprofessional opportunities, and who had positive views of this type of collaboration. Seventeen of the 26 participants noted that they had some ‘prior interprofessional experience’. Additionally, there was no blinding, as all of the participants fully understood the project objectives. Surveys were completed at the beginning and end of each summer project, when awareness of teamwork and communication issues was likely highest in students’ minds. Of interest to note is that only three males participated in the *VIP-CARES Project* over the three years of this study, which could reflect a gendered preference for the topic of family planning among prospective participants. In addition, the disciplines involved in the project (i.e. midwifery, nursing, pharmacy, and medicine) may have also contributed to the skewed gender ratio, as all of these programs at UBC have a higher proportion of female enrollment. Previous studies have also examined gender as a factor affecting attitudes towards IPC [26]. Future similar studies on IPC and attitudes may wish to explore methods to sample a more balanced gender mix.

## Conclusion

This mixed methods study evaluated the changes in perception towards interprofessional collaboration (IPC) among pre-licensure health professional students before and after working on the *VIP-CARES Project*. Students participated in an eight-week interdisciplinary team, developing virtual patient case-based learning modules on the topic of family planning. The findings from the surveys were supported by the themes elicited from the qualitative data. Attitudes towards IPC were positive amongst students before and after participating in *VIP-CARES*. At the project’s conclusion, there was a statistically significant increase in the participants’ self-assessment competency scores in the CanMEDS roles of *health advocate*, *manager*, and *medical expert*, as well as the CIHC domains of *interprofessional communication*, *role clarification*, *team functioning*, and *collaborative leadership*. From the oral and written reflections, the students generally felt more comfortable with their improvements in the CIHC domains of *interprofessional communication*, *team functioning*, and *role clarification*.

## Additional file

Additional file 1: Self-evaluation questionnaire: Evaluating health professional interprofessional collaboration and attitudes. This questionnaire is the instrument used in this study. (PDF 113 kb)
