# Supplementary material for: Measuring interprofessional competencies and attitudes among health professional students creating family planning virtual patient cases
Source: BMC Med Educ. 2016 Oct 19;16:273. doi: 10.1186/s12909-016-0797-8 (PMC5069921; doi:10.1186/s12909-016-0797-8)
Supplement: Additional file 1: — Self-evaluation questionnaire: Evaluating health professional interprofessional collaboration and attitudes. This questionnaire is the instrument used in this study. (PDF 113 kb) [file 12909_2016_797_MOESM1_ESM.pdf]

a place of mind
THE UNIVERSITY OF BRITISH COLUMBIA

CART Contraception & Abortion Research Team

SELF-EVALUATION QUESTIONNAIRE

Evaluating health professional interprofessional collaboration and attitudes

This Questionnaire is available for use with permission of the authors:

Eric Wong MD, Jasmine J. Leslie MD, Judith A. Soon R. Pharm, PhD (Pharm), * Wendy V. Norman MD, MHSc
University of British Columbia, Vancouver, British Columbia

***Correspondence:**

Dr. Wendy V. Norman,
Associate Professor, Department of Family Practice, University of British Columbia,
3rd Floor, David Strangway Building, 5950 University Boulevard, Vancouver, BC, V6T 1Z3, Canada.
E-mail: wendy.norman@ubc.ca

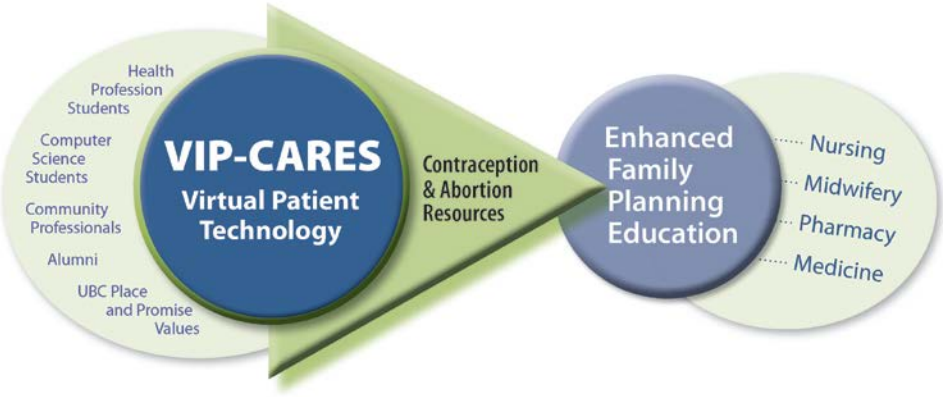

A. Background

First, please answer the following background questions. All surveys are anonymous and group summaries of results only will be reported.

1. To correctly link your data, please provide your name:

   ____

2. In which health professional program area and year are you currently registered:

   Name of Program: ________________________________

   Year(s) of this program I have completed: ________________

   Total Number of Years in this program: _________________

3. What is your **year** of birth (e.g. 1978)? ______________

4. Are you: □ female □ male

5. Have you had any **prior** involvement with interprofessional education activities? (please check all that apply)

   □ Interprofessional Education I (COPC module)
   □ Interprofessional Education II (HIV/AIDS module)
   □ Clinical/practice experiences in settings with interprofessional teams

   □ Other (please specify) ____

6. What is your current student status?

   □ Full-time
   □ Part-time

B. Attitudes towards interprofessional health care teams and education

We are interested in learning how you feel about interprofessional health care teams (i.e. participation of three or more professions in collaborative patient care) and interprofessional education (i.e. shared learning activities involving students from more than one health care professional program).

Please indicate your level of agreement with each of the following statements, by checking the appropriate space following each statement.
Use the scale SD = strongly disagree; D = disagree; N = neutral; A = agree; SA = strongly agree.

STATEMENT:	SD	D	N	A	SA
7. Interprofessional learning will help students to understand their own professional limitations.					
8. Developing an interprofessional patient/client care plan is excessively time consuming.					
9. The interprofessional approach makes the delivery of care more efficient.					
10. Developing a patient/client care plan with other team members avoids errors in delivering care.					
11. Working in an interprofessional manner unnecessarily complicates things most of the time.					
12. The interprofessional approach improves the quality of care to patients/clients.					
13. In most instances, the time required for interprofessional consultations could be better spent in other ways.					
14. The interprofessional approach permits health professionals to meet the needs of family caregivers as well as patients.					
15. Team meetings foster communication among team members from different professions or disciplines.					
16. Interprofessional learning will help students think positively about other health care professionals.					
17. Clinical information can only be learned effectively when students are taught within their individual department/school.					
18. Students in my professional group would benefit from working on small group projects with other health care students.					
19. It is not necessary for undergraduate health care students to learn together.					
20. Learning between health care students before qualification would improve working relationships after qualification.					
21. Learning with students in other health professional schools helps undergraduates to become more effective team members.					

C. CanMEDS Professional Competencies

We are interested in learning how you rank yourself as a professional according to the following CanMEDS roles. Please note that the bullet points are not exhaustive of the different roles, merely representatives of what may be included under each heading.

Please indicate your self-rating of each of the following roles, by circling ONLY ONE of the appropriate number below each rating. Feel free to make comments as you feel necessary.

CanMEDS Role:	Below Expectations		Borderline		Meets Expectations		Exceeds Expectations		Exceptional	
**22. Communicator**	1	2	3	4	5	6	7	8	9	10
- Obtain relevant information for optimal care<br>- Effective listening skills (empathy, accuracy, etc)<br>- Excellent verbal and non-verbal communication<br>- Address and discuss "difficult" topics<br>- Clear, thorough records & notes	Identify strength(s)/ area(s) to improve:									
**23. Collaborator**	1	2	3	4	5	6	7	8	9	10
- Share decision making with other professionals<br>- Engage and consider patient's support people<br>- Respectful of group diversity when working in teams<br>- Constructive conflict-resolution & prevention<br>- Maintain positive working environment	Identify strength(s)/ area(s) to improve:									
**24. Health Advocate**	1	2	3	4	5	6	7	8	9	10
- Advocate for individuals, populations & communities<br>- Promote patient health and disease prevention<br>- Consider the determinants of health (psychological, physical, cultural, economical, barriers to health care)<br>- Identify at-risk or marginalized populations	Identify strength(s)/ area(s) to improve:									
**25. Manager**	1	2	3	4	5	6	7	8	9	10
- Appropriate allocation of finite healthcare resources<br>- Effective priority-setting, time management<br>- Use of information technology for health care<br>- Clear understanding of roles and responsibilities within health care system	Identify strength(s)/ area(s) to improve:									

CanMEDS Role:	Below Expectations		Borderline		Meets Expectations		Exceeds Expectations		Exceptional	
**26. Medical Expert**	1	2	3	4	5	6	7	8	9	10
- Integrate ALL the CanMEDS roles<br>- Choose appropriate therapies/solutions/answ ers<br>- Medical know ledge and skills relevant to your field<br>- Patient-centered decision making<br>- Understand, demonstrate patient safety & protection<br>- Know your limits of expertise	Identify strength(s)/ area(s) to improve:									
**27. Professional**	1	2	3	4	5	6	7	8	9	10
- Altruism, integrity, honesty, compassion and caring<br>- Commitment to high level of professional standards (on time, respect, use of appropriate language/dress)<br>- Commitment to ethical practice<br>- Disclose of errors/ mistakes/ misunderstandings, etc<br>- Self-reflection	Identify strength(s)/ area(s) to improve:									
**28. Scholar**	1	2	3	4	5	6	7	8	9	10
- Identify gaps in know ledge<br>- Ask effective questions to fill in gaps<br>- Facilitate learning of others (incl. patients)<br>- Ongoing self-directed learning<br>- Critical evaluation of resources/information	Identify strength(s)/ area(s) to improve:									

D. CIHC Interprofessional Competencies

We are interested in learning how you rank yourself as a professional according to the following Canadian Interprofessional Health Collaborative (CIHC) competencies. Please note that the bullet points are not exhaustive of the different roles, merely representatives of what may be included under each heading.

Please indicate your self-rating of each of the following roles, by circling ONLY ONE of the appropriate number below each rating. Feel free to make comments as you feel necessary.

CIHC Interprofessional Competency	Below Expectations		Borderline		Meets Expectations		Exceeds Expectations		Exceptional	
**29. Interprofessional Communication**	1	2	3	4	5	6	7	8	9	10
-communicate with a varied array of professionals<br>- responsive, responsible and respectful in communication with other professionals.	Identify strength(s)/ area(s) to improve:									
**30. Patient-Centered Care**	1	2	3	4	5	6	7	8	9	10
- Seek out, integrate, engage and respect patient/client/ family/community in creating health care plans.	Identify strength(s)/ area(s) to improve:									
**31. Role Clarification**	1	2	3	4	5	6	7	8	9	10
- understand your own role and the roles of other professionals<br>- use this knowledge to enhance patient/client/family and community goals.	Identify strength(s)/ area(s) to improve:									
**32. Team Functioning**	1	2	3	4	5	6	7	8	9	10
- Understand team dynamics and enhance collaboration between professionals in a team.	Identify strength(s)/ area(s) to improve:									
**33. Collaborative Leadership**	1	2	3	4	5	6	7	8	9	10
- Work together with ALL participants (including patients/clients/families) to create care plans<br>- Follow through with ALL participants on implementing and evaluating care plans.	Identify strength(s)/ area(s) to improve:									
**34. Interprof. Conflict Resolution**	1	2	3	4	5	6	7	8	9	10
- Actively engage in dealing with interprofessional conflict.<br>- Actively engage others (including the patient/client/ family in dealing with such conflict.	Identify strength(s)/ area(s) to improve:									
